# Host-level biodiversity shapes the dynamics and networks within the coral reef microbiome

**DOI:** 10.1093/ismeco/ycaf097

**Published:** 2025-06-05

**Authors:** Fabienne Wiederkehr, Kara Elena Engelhardt, Jana Vetter, Hans-Joachim Ruscheweyh, Guillem Salazar, James O’Brien, Taylor Priest, Maren Ziegler, Shinichi Sunagawa

**Affiliations:** Department of Biology, Institute of Microbiology and Swiss Institute of Bioinformatics, ETH Zürich, 8093 Zürich, Switzerland; Department of Animal Ecology & Systematics, Justus Liebig University Giessen, 35392 Giessen, Germany; Department of Animal Ecology & Systematics, Justus Liebig University Giessen, 35392 Giessen, Germany; Department of Biology, Institute of Microbiology and Swiss Institute of Bioinformatics, ETH Zürich, 8093 Zürich, Switzerland; Department of Biology, Institute of Microbiology and Swiss Institute of Bioinformatics, ETH Zürich, 8093 Zürich, Switzerland; Department of Biology, Institute of Microbiology and Swiss Institute of Bioinformatics, ETH Zürich, 8093 Zürich, Switzerland; Department of Biology, Institute of Microbiology and Swiss Institute of Bioinformatics, ETH Zürich, 8093 Zürich, Switzerland; Department of Animal Ecology & Systematics, Justus Liebig University Giessen, 35392 Giessen, Germany; Department of Biology, Institute of Microbiology and Swiss Institute of Bioinformatics, ETH Zürich, 8093 Zürich, Switzerland

**Keywords:** biodiversity, coral reef, microbiome, holobiont, metacommunity

## Abstract

Coral reefs face severe threats from human activity, resulting in drastic biodiversity loss. Despite the urgency of safeguarding these ecosystems, we know little about the ecological impacts of losing coral reef host–associated microbial communities (microbiomes). Here, we experimentally studied the microbiomes attached to or released from seven benthic reef hosts belonging to the functional groups of stony corals, soft corals, macroalgae, and sponges while manipulating the coral reef metacommunity to mimic biodiverse or degraded reef habitats. Developing an ecological framework, we found host species and functional groups to show distinct patterns of interacting with the environment (i.e. by exuding, maintaining, acquiring, or exchanging microbiome members), with habitat biodiversity primarily influencing microbial acquisition. In a degraded compared to a biodiverse habitat, the microbiomes of stony corals were less connected to soft corals and sponges, while those of soft corals, macroalgae, and sponges became more tightly linked. Our study demonstrates that a decline in metacommunity biodiversity is not merely associated with a proportional loss in microbial diversity; rather, it triggers complex changes in the microbial interactions among the persisting hosts with each other and the environment. These results emphasize the importance of conserving coral reef host biodiversity to preserve the intricately linked microbiomes—and with them the ecosystem functions and services coral reefs provide.

## Introduction

Coral reefs are ecologically, culturally, and economically important ecosystems. They are highly diverse and productive, providing invaluable services such as food, livelihoods, carbon sequestration, and coastal protection [1]. These reefs, built by stony corals, are shaped by a diverse community of benthic reef organisms spanning multiple functional groups, including soft corals, macroalgae, and sponges [2]. Due to the combined pressures of climate change, emerging diseases, and local stressors, coral reefs have undergone drastic changes: live coral cover has decreased by >50% during the past 70 years [1, 3], resulting in a shift from stony corals (the reef builders) to other functional groups in many benthic reef communities [4–6]. Stony-coral-dominated reefs may cease to exist with as little as 1.2°C warming [7], highlighting the urgent need to comprehend the ecological consequences of these community shifts and the resulting biodiversity loss—beyond the level of macroorganisms.

Central to all macroorganisms (hosts) of coral reefs is a diverse and species-specific community of microorganisms (microbiome) [8–13], collectively forming the holobiont. The microbiome supplies the hosts with essential nutrients, vitamins, and amino acids [14, 15], playing a crucial role in the health and functioning of coral reef hosts [2, 16]. By mediating processes such as nutrient cycling and stress response, the coral reef microbiome is pivotal in the fight against climate change, helping the hosts and ecosystem to adapt to environmental pressures [17, 18]. Achieving a balance between microbiome flexibility and stability is crucial for the ecological success and environmental resilience of coral reef holobionts [19, 20]. To effectively utilize the power of microbiomes and promote reef conservation [21], we need to deepen our understanding of the dynamic interactions between the hosts and their associated microbiomes.

Bridging the gap between micro- and macro-scales is particularly important in the face of biodiversity loss. A microbiome perspective on coral reefs may offer insights into how host-associated microbiomes impact the coral reef metacommunity and how this potentially feeds back on the hosts [22–24]. For instance, the microbiome of corals competing with macroalgae may shift due to macroalgae exudates that support copiotrophic and potentially pathogenic microorganisms in the water column, which may trigger coral disease and mortality, making more space available to macroalgae and increasing the competitive pressure on corals [22, 25]. While studies from the natural environment provide valuable hypotheses for the microbial dynamics within coral reefs, we lack controlled experiments [26] and a mechanistic framework [27] to disentangle how coral reef hosts interact microbially—with the environment and each other.

To this end, we studied seven sessile coral reef hosts belonging to stony corals, soft corals, macroalgae, and sponges within a controlled experimental system. Based on the host-associated (attached to the host), exuded (released from the host), and free-living (surrounding the host) microbiomes, we designed an ecological framework to disentangle different processes by which holobionts interact with each other and the environment, specifically by maintaining, exuding, acquiring, and exchanging microbiome members. Given the alarming decline in reef cover, we examined how these links and processes change in response to different levels of complexity in the coral reef metacommunity. We demonstrate that microbial dynamics are species-specific and that habitat complexity shapes the microbial links between coral reef holobionts. Our study highlights the importance of expanding research, conservation, and restoration efforts beyond individual holobionts to adopt a metacommunity perspective [23], accounting for an interconnected microbiome network to maintain or restore healthy coral reef ecosystems.

## Materials and methods

### Experimental procedure

**Figure 1:**
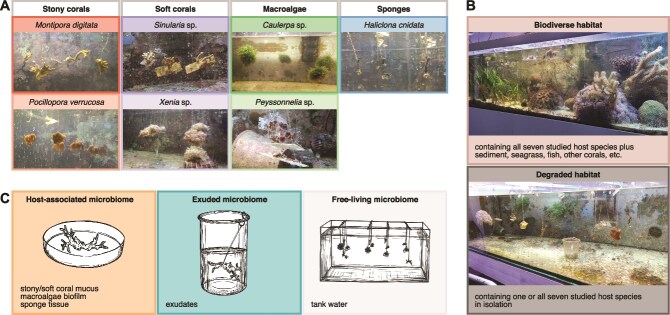
Experimental setup and sampling design. (A) We studied seven sessile coral reef hosts belonging to the functional groups of stony corals (*M. digitata*, *P. verrucosa*), soft corals (*Sinularia* sp., *Xenia* sp.), macroalgae (*Caulerpa* sp., *Peyssonnelia* sp.), and sponges (*H. cnidata*) within a controlled experimental aquarium. (B) Given the alarming decline in reef biodiversity, we examined how the microbial communities influence and are influenced by a biodiverse (i.e. a reef community including sediments and a diverse range of reef organisms) and a degraded (i.e. one or all seven studied host species kept in isolation) habitat. (C) We sampled the host-associated and exuded microbial communities associated with these hosts as well as the free-living microbial community of the ambient tank water.

In a 3-month experiment (Suppl. Fig. 1A) conducted at the *Ocean2100* aquarium facility [28] at Justus Liebig University Giessen, Germany, we investigated the microbiome dynamics of seven coral reef hosts (Fig. 1A): the stony corals *Montipora digitata* (Dana, 1846) and *Pocillopora verrucosa* (Ellis & Solander, 1786), the soft corals *Sinularia* sp. (May, 1899) and *Xenia* sp. (Lamarck, 1816), the macroalgae *Caulerpa* sp. (Lamouroux, 1809) and *Peyssonnelia* sp. (Decaisne, 1841), and the sponge *Haliclona cnidata* (Schellenberg, Reichert, Hardt, Schmidtberg, Kämpfer, Glasser, Schubert & Wilke, 2019). These host species were selected based on their photosynthetic productivity in response to proximity to other reef organisms [29, 30], and the functional groups are ecologically linked [31], fulfilling pivotal roles in retaining and recycling nutrients within coral reefs [32, 33] and thereby shaping ecosystem functioning.

To examine the effects of host-level biodiversity on microbial dynamics, we manipulated the complexity of the habitat in our experimental system (Fig. 1B). To mimic a species-rich and diverse coral reef, we chose an aquarium that harboured (in addition to the seven studied species) sediment, seagrass, fish, and an assortment of benthic reef organisms, most of which were stony corals (herein referred to as a biodiverse habitat). We contrasted this to a species-poor environment in which our studied species lived in isolation (herein referred to as a degraded habitat). This design allowed us to assess how the loss of biodiversity influences the composition of and dynamics within the coral reef microbiome.

Host fragments (*n* = 15) were prepared, allowed to heal, and maintained as described in Engelhardt *et al*. [34]. Briefly, the fragments were suspended contact-free in the middle of the water column and kept at a temperature of 26°C, light intensity of 200 ± 20 μmol photons m^−2^ s^−1^, and salinity of 35 psu. After a 46-day acclimatization period, during which all fragments (*n* = 105) were maintained in tanks of degraded complexity, one subset of fragments (*n* = 63) remained in these tanks, while the remaining fragments (*n* = 42) were transferred into the biodiverse tank.

### Sample collection

To characterize the coral reef microbiome, we collected three types of samples (Fig. 1C). Firstly, we sampled the microbial communities attached to the hosts (host-associated microbiome; Materials and Methods). Specifically, we collected mucus of stony and soft corals, biofilm of macroalgae, and tissue of sponges (i.e. the parts of the hosts that are exposed to and interact with the environment). Secondly, we collected the microbial communities that the hosts released during a 2-h incubation period (exuded microbiome; Materials and Methods; Suppl. Fig. 1B) into filtered tank water (control microbiome). Lastly, we filtered the microbial communities of the tank water (free-living microbiome; Materials and Methods).

We collected samples at three time points: after 10–13 days (phase 0), after 37–40 days (phase 1), and after 64–67 days (phase 2, 18–21 days after transferring to the respective habitat). Firstly, we collected the free-living microbiome (500 ml of tank water) from each tank in triplicate on 0.22 μm Sterivex filters. Secondly, we collected the microbiome exuded into the surrounding water (incubations). To prepare water for the exuded microbiome incubations, we sequentially filtered artificial seawater (ASW) from an empty tank within the same aquarium system through a 65 μm mesh net, followed by 0.45, 0.22, and 0.2 μm pore-sized filters. We poured 600–650 ml of the sterile-filtered ASW into sterile 1 l glass jars. We then collected the fragments from the tanks, rinsed them with sterile-filtered ASW, and placed them in the incubation jars for 2 h on a multipoint stirring plate [35] at 9 ± 1.5 cm s^−1^ flow and 200 ± 20 photons μmol m^−2^ s^−1^ (Suppl. Fig. 1B). This exuded microbiome (as well as control microbiomes from jars without fragments) was collected on 0.22 μm Sterivex filters. Thirdly, immediately after the incubations, we collected host-associated samples. Specifically, we air-exposed stony and soft coral fragments to obtain mucus (milking [36]) and collected macroalgae and sponge tissue. All samples were flash-frozen in liquid nitrogen and stored at −80°C until further processing.

### Sample processing and DNA extraction

For host-associated samples, DNA was extracted using the DNeasy PowerBiofilm kit (Qiagen). For coral mucus, we added 100 μl of mucus directly to the bead-beating tubes. For macroalgae, we separated the macroalgal biofilm from the tissue by incubating the tissue in 5 ml of sterile 1× PBS for 18 h at 200 rpm and 37°C [24]. We transferred the supernatant and centrifuged (14 000 × *g*, 15 min, 4°C), discarded the supernatant, and resuspended the pellet in 100 μl of buffer MBL (first buffer used during extractions) and added this to the bead-beating tubes. For sponges, we homogenized the tissue in 5 ml of sterile Tris–HCl-NaCl buffer on ice for 3 min using the Polytron PT 1200 E with the aggregate PT-DA 07/2SYN-E082 at medium speed [37] and added 100 μl of the homogenate directly to the bead-beating tubes. The bead-beating was performed using the Retsch Mixer Mill MM400 (5 min at 25 Hz, re-orienting tubes, 5 min at 25 Hz). No other modifications were made, and we eluted in 100 μl of EB.

For Sterivex samples (exuded, control, and tank microbiomes), we extracted the DNA using the DNeasy PowerWater Sterivex kit (Qiagen) and eluted in 60 μl of EB.

### Amplicon library preparation and sequencing

To prepare the samples for amplicon sequencing, we generated our libraries in a two-step polymerase chain reaction (PCR) initial denaturation of 98°C for 30 s followed by cycles of 98°C for 10 s, 56°C for 30 s, and 72°C for 30 s, and a final extension of 72°C for 2 min. In the first PCR, we amplified the 16S ribosomal RNA (rRNA) gene with primers 515F-Y (5′-GTGYCAGCMGCCGCGGTAA [38]) and 806R (5′-GGACTACNVGGGTWTCTAAT [39]) in 50 μl of Q5 (BioConcept) PCR reactions (10 μl of DNA template added to 10 μl of 5× Q5 Reaction Buffer, 10 μl of 5× Q5 High GC Enhancer, 0.5 μl of Q5 High-Fidelity DNA Polymerase (2000 U ml^−1^), 1 μl of 10 mM deoxynucleoside triphosphates (dNTPs) (Thermo Scientific), 2.5 μl of 10 μM forward primer, 2.5 μl of 10 μM reverse primer, 13.5 μl of nuclease-free water), and 20 cycles. After clean-up (0.8× Sera-Mag Select; Cytiva), we performed the second PCR with barcoding primers containing Illumina’s unique dual index adapters in 25 μl of Q5 (BioConcept) PCR reactions (11 μl of PCR1 product, 5 μl of 5X Q5 Reaction Buffer, 5 μl of Q5 High GC Enhancer, 0.25 μl of Q5 High-Fidelity DNA Polymerase (2000 U ml^−1^), 0.5 μl of 10 mM dNTPs (Thermo Scientific), 2.5 μl of 10 μM barcoding primers, 0.75 μl of nuclease-free water), and 15 cycles. After clean-up (0.7× Sera-Mag Select; Cytiva), we quantified the PCR products and pooled the libraries equimolarly.

Using a 250 bp paired-end approach at the Genome Engineering and Measurement Lab at ETH Zürich, all host-associated samples were sequenced on the Illumina MiSeq platform with 25% PhiX (V3; Illumina) and all Sterivex samples on the Illumina NextSeq platform with 40% PhiX (V3; Illumina).

### Data generation

We processed the 844 metabarcoding samples using the procedures described in https://github.com/SushiLab/IMB_Amplicon_Pipeline. In brief, primer sequences were removed using cutadapt (v3.5) [40] and only paired reads that contained both primers (-O 12, −-discard-untrimmed, −-minimum-length 75) were kept for downstream analysis. Next, reads from Sterivex samples were quality-filtered using the filterAndTrim function of the dada2 (v1.22) [41] package [rm.phix = TRUE, maxEE = 2, truncQ = 2, truncLen = c(191,151)]. Quality control of sequencing reads from host-associated samples was performed as for Sterivex samples but varied in the truncLen parameter due to decreased quality of the reverse reads [truncLen = c(201,81) and truncLen = c(161,121)]. We used the functions learnErrors (nbases = 1e7, randomize = TRUE, errorEstimationFunction = loess_error_function_mod) to calculate error estimates for nucleotide transition probabilities and dada (pool = pseudo) to calculate sample inference and infer amplicon sequence variants (ASVs).

Reads were merged using the function mergePairs and bimeras were removed with removeBimeraDenovo (method = pooled). The remaining ASVs were taxonomically annotated using IDTAXA (v2.22) [42] and the SILVA database (v138) [43].

### Statistics and reproducibility

Statistical analyses were performed in R (v4.2.2–4.4.0). We removed all reads unclassified at the domain level, belonging to the domain Eukaryota, the family Mitochondria, or the order Chloroplast, as well as singleton ASVs (present in a single sample with an abundance of 1). Using the R package decontam (v1.24.0) [44], we explored various thresholds using frequency and prevalence probabilities combined with Fisher’s method to identify contaminants and selected 0.1 (default) for the host-associated microbiome data and 0.2 (more aggressive) for the exuded and free-living microbiome data. We merged the two decontaminated datasets and taxonomically annotated the sequences using the R package dada2 (v1.32.0) [41] and the SILVA database (v138.1) [43]. For global analyses, we used all data, while for comparisons between habitat complexities, we used the data from Phase 2 only.

To account for unequal sequencing effort (Suppl. Fig. 2), we rarefied the data 50 times to 2500 reads per sample using the R package vegan (v2.6–8). Metrics were computed for each of the 50 rarefied datasets, and the mean value of each metric was used in the analyses [45]. Beta diversity was calculated as Bray–Curtis dissimilarities [46] based on square-root-transformed abundance data, which mitigates the influence of highly abundant species [47]. For alpha diversity, we computed Hill numbers [48] of orders *q* = 0 (species richness, weighing all ASVs equally), *q* = 1 (exponential of Shannon’s entropy index, weighing ASVs proportionally to their frequency), and *q* = 2 (inverse of Simpson’s concentration index, emphasizing abundant ASVs) [49] using the R package hillR (v0.5.2) [50]. The number of shared ASVs was calculated using vegan’s designdist (method = “J” and terms = “binary”), while the number of unique ASVs for each host species was determined with the R package ComplexUpset (v1.3.3) [51, 52]. For the networks, we calculated the ratio between biodiverse and degraded habitats as: 0.5–*x*_degraded_ × (*x*_degraded_ + *x*_biodiverse_)^−1^, with *x* being similarity, richness, number of shared ASVs, or number of unique ASVs. Networks were generated using the R packages igraph (v2.0.3), tidygraph (v1.3.1), and ggraph (v2.2.1). Comparisons between groups were analysed using the Wilcoxon rank-sum, Permutational Multivariate Analysis of Variance (PERMANOVA), or Multivariate Analysis of Variance (MANOVA) tests as appropriate, using the R packages stats (v4.4.0), vegan (v2.6–8), and pairwiseAdonis (v0.4.1). When applicable, multiple testing corrections were applied using the Holm method. Visualizations were created with ggplot2 (v3.5.1).

## Results

### Disentangling dynamic processes within the coral reef microbiome

**Figure 2:**
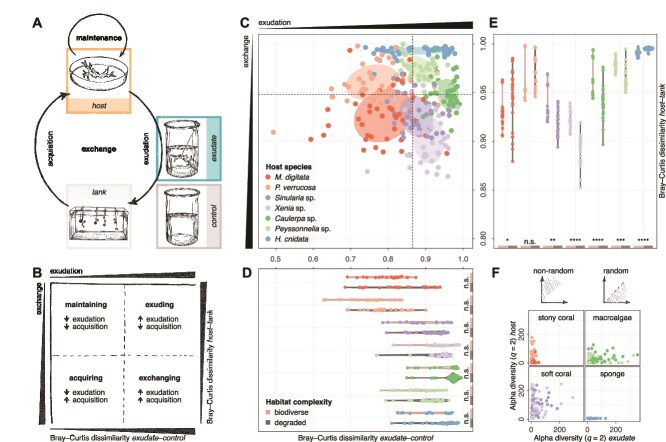
Species-specificity and influence of the ambient community complexity on the microbial exchange. (A) We developed a theoretical framework to study how coral reef hosts interact with the environment (*host*: Host-associated microbiome; *exudate*: Exuded microbiome; *tank*: Free-living microbiome in the ambient tank water; *control*: Filtered tank water and baseline of the exudate samples) (Materials and Methods). (B) By comparing exudation (Bray–Curtis dissimilarities between *exudate* and *control* samples) and exchange (Bray–Curtis dissimilarities between *host* and *tank* samples), we inferred acquisition and differentiated different processes by which holobionts interact with the environment (i.e. by exuding, maintaining, acquiring, or exchanging; Materials and Methods). (C) The dynamics within the coral reef microbiome were species-specific (MANOVA, Pillai’s trace = 1.250, *F*_12 564_ = 78.26, *P* < .001; all pairwise comparisons significant, *P* < .001) and clustered the hosts by functional group (MANOVA, Pillai’s trace = 1.054, *F*_6570_ = 105.87, *P* < .001; all pairwise comparisons significant, *P* < .001). (D) Exudation was not influenced by the complexity of the reef community; however, (E) exchange was (except for *P. verrucosa*). In a biodiverse reef community, the microbiomes of the two soft corals *Sinularia* sp. (Wilcoxon test, *W* = 302, adjusted *P*-value = .003) and *Xenia* sp. (Wilcoxon test, *W* = 469, adjusted *P*-value < .001) and the two macroalgae *Caulerpa* sp. (Wilcoxon test, *W* = 348, adjusted *P*-value < .001) and *Peyssonnelia* sp. (Wilcoxon test, *W* = 358, adjusted *P*-value < 0.001) exchanged less (i.e. the microbiomes of *host* and *tank* were more dissimilar), whereas the microbiome of the stony coral *M. digitata* (Wilcoxon test, *W* = 145, adjusted *P*-value = .046) and the sponge *H. cnidata* (Wilcoxon test, *W* = 80, adjusted *P*-value < 0.001) exchanged more (i.e. the microbiomes of host and tank were less dissimilar). (F) Correlating the alpha diversity (Hill *q* = 2) of the exuded and host-associated microbiomes suggested corals retain abundant microbial community members, whereas macroalgae and sponges exude them. Statistical significance is indicated as follows: n.s. (*P*-value ≥ .05), ^*^(*P* < .05), ^**^(*P* < .01), ^***^(*P* < .001), ^****^(*P* < .0001).

To understand how coral reef holobionts interact with the environment, we developed a theoretical framework based on our experimental setup (Fig. 2A). We postulated that coral reef holobionts maintain a portion of their microbiome and exchange the rest—be that actively or passively, and driven by environmental factors, the host, or the microbiome itself [53]. The exchanged microbiome is a composite of microorganisms released into (exuded) or taken up from (acquired) the environment. In this study, we described microbial exchange (≥0.22 μm) by comparing the host-associated and free-living microbiomes (Bray–Curtis dissimilarities; Materials and Methods), represented on the *y*-axis, where high dissimilarity indicates low rates of microbial exchange (Fig. 2B). Microbial exudation was assessed by comparing the exuded and control microbiomes (Bray–Curtis dissimilarities; Materials and Methods), shown on the *x*-axis, where high dissimilarity reflects high rates of microbial exudation (Fig. 2B). Finally, we inferred microbial acquisition by examining the relationship between exudation and exchange, considering exchange as the composite of exudation and acquisition (Fig. 2B). Using this framework, we defined four key processes shaping coral reef microbiome dynamics: exuding (characterized by high exudation and low acquisition), maintaining (characterized by low exudation and low acquisition), acquiring (characterized by low exudation and high acquisition), and exchanging (characterized by high exudation and high acquisition).

This framework effectively enabled us to disentangle these processes in our studied coral reef holobionts relative to one another and investigate species-specific patterns of microbiome exchange. Indeed, we observed that the dynamics within the coral reef microbiome were specific to each species (MANOVA, Pillai’s trace = 1.250, *F*_12 564_ = 78.26, *P* < .001) but similar within the functional group (MANOVA, Pillai’s trace = 1.054, *F*_6570_ = 105.87, *P* < .001) (Fig. 2C; Suppl. Fig. 3A), a pattern shared with the composition (Suppl. Fig. 3B; Suppl. Table 1) and predicted functional profiles (Suppl. Fig. 3C) of the coral reef microbiome. Although varying in the levels of exudation, the sponge’s host-associated microbiome was most dissimilar to the tank water, which is consistent with the reported stability of the sponge microbiome [54, 55] and possibly related to our sampling strategy as sponge samples included tissue-associated microorganisms. The stony corals *M. digitata* and *P. verrucosa* exhibited the lowest exudation levels, suggesting that they contributed the least to the microbial pool available in the environment. In contrast, the soft corals *Sinularia* sp. and *Xenia* sp. and the macroalgae *Caulerpa* sp. and *Peyssonnelia* sp. showed high rates of exudation but differed in rates of acquisition, which was high for the two soft corals and low for the two macroalgae. The observed differences in acquisition between the soft corals and macroalgae might result from heterotrophic feeding by soft corals to meet their nutrient requirements [56, 57]. The difference in exudation between the stony and soft corals may indicate that the latter group releases more particulate organic matter [58]. By calculating the mean values for exudation and exchange (0.865 and 0.948, respectively; dashed lines in Fig. 2C), we found that overall and relative to one another, the stony corals *M. digitata* and *P. verrucosa* were acquiring and maintaining, respectively, the soft corals *Sinularia* sp. and *Xenia* sp. exchanging, the macroalgae *Caulerpa* sp. and *Peyssonnelia* sp. exuding, and the sponge *H. cnidata* exuding or maintaining. While the observed variation in the sponge’s strategy raises intriguing questions about the factors driving this flexibility, our results underscore the distinct ecological roles the functional groups play in sustaining coral reef ecosystem functioning.

Next, we investigated how these strategies were affected by the complexity of the reef metacommunity. We found that, while reef community complexity did not influence exudation (Fig. 2D), it did affect exchange (except for the maintaining stony coral *P. verrucosa*, which is known to host a stable microbiome [59, 60]) (Fig. 2E). Since exudation remained the same, we concluded that the differences in exchange were driven by acquisition. We observed that the microbiomes of the stony coral *M. digitata* and the sponge *H. cnidata* acquired more microorganisms in a biodiverse environment compared to a degraded environment, suggesting that increased habitat complexity promotes acquisition in these hosts. Conversely, soft corals and macroalgae acquired fewer microorganisms in a biodiverse habitat.

To evaluate how nonrandom microbial exudation by coral reef holobionts is, we correlated the richness and diversity of the microbiomes associated with and released from the hosts. If exudation were nonrandom, host-associated microbiomes would be richer/more diverse (points distributed along the *y*-axis) than the exuded microbiomes (Fig. 2F). Conversely, random exudation would result in equally (or more) rich/diverse exuded microbiomes (points distributed along the *x*-axis) (Fig. 2F). Our analysis revealed that exuded microbiomes were more diverse than their host-associated counterparts in the macroalgae *Caulerpa* sp. and *Peyssonnelia* sp., as well as the sponge *H. cnidata* (Fig. 2F; Wilcoxon rank-sum tests, *P*-value < .05; Suppl. Table 2). In contrast, in the stony coral *P. verrucosa* and the soft corals *Sinularia* sp. and *Xenia* sp., exuded microbiomes were less diverse than those associated with the hosts (Fig. 2F; Suppl. Fig. 4A; Wilcoxon rank-sum tests, *P*-value < .05; Suppl. Table 2). Despite these differences in diversity, microbial richness was consistently higher in the exuded microbiomes across hosts (Suppl. Fig. 4A; Wilcoxon rank-sum tests, *P*-value < .05; Suppl. Table 2), showing that the abundance of the microbial community members is relevant for the observed differences. Together, the findings suggest that corals nonrandomly retain abundant microbiome members, which may contribute to their microbiome stability [18, 61]. In contrast, the surplus of microbial diversity in macroalgae and sponge exudates might stem from continuous host cell shedding [31, 62], which releases tissue-associated microorganisms, and, in the case of sponges, active or passive removal of particulate waste from their bodies [63]. These processes likely contribute to the observed differences in microbial (dis)similarity between host-associated and exuded microbiomes (Suppl. Fig. 4B), reinforcing the relevance of alpha diversity metrics in capturing patterns of nonrandom microbial exudation.

### Mapping interaction networks within the coral reef microbiome

To examine how the microbial dynamics of individual holobionts impact the coral reef metacommunity, we compared the host-associated and exuded microbiomes between hosts. The similarities within the exuded microbiomes were higher than within the host-associated microbiomes (Suppl. Fig. 5; Suppl. Table 3), indicating that, in addition to maintaining host-specific microbiomes [9], holobionts interact transiently with numerous non-host-associated microorganisms. In particular, we found the sponge *H. cnidata* to be more connected to other hosts in the exuded microbiome, which aligns with its ecological role as filter feeder and resource recycler [31, 63].

**Figure 3:**
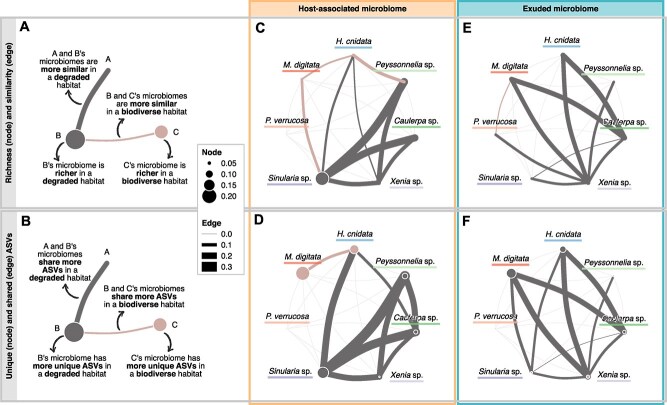
Differential microbial links between coral reef holobionts depending on the ambient community complexity. We compared the changes in microbiome (A, C, E) similarity between (edges; Bray–Curtis dissimilarity) and richness of (nodes; Hill *q* = 0) and numbers of (B, D, F) shared ASVs between (edges) and unique ASVs of (nodes) coral reef holobionts due to habitat complexity (Suppl. Fig. 5). Colours indicate the direction of change and node size/edge width the magnitude (only significant changes are shown, except for the number of shared ASVs; Suppl. Table 4). Values were calculated as the difference to the mean (i.e. no change; Materials and Methods). 0.2 translates to a 40% difference between biodiverse and degraded habitats. (A, B) The schematics serve as an example of how to interpret the networks. (C) For the host-associated microbiomes, we found soft corals and macroalgae to be more similar in a degraded habitat and the stony coral *M. digitata* and the sponge *H. cnidata* to be more similar in a biodiverse habitat. (D) Similarly, we found soft corals and macroalgae to share more ASVs in a degraded habitat. The sponge’s connectivity to other hosts was stronger from a microbial member than community perspective. (E) For the exuded microbiomes, we found all holobionts (except for the stony corals) to be more similar in a degraded habitat. (F) Likewise, ignoring abundances showed that the holobionts shared more ASVs in a degraded habitat.

Next, we explored how the connectivity within the coral reef microbiome changes in response to the complexity of the habitat. We employed both a microbial community perspective (similarity and richness) (Fig. 3A) and a microbial member perspective (shared and unique ASVs) (Fig. 3B), allowing for a nuanced interpretation of these changes. For the host-associated microbiomes, the changes in similarities between holobionts (Fig. 3C) seemingly reflect changes in host–environment interactions caused by habitat complexity. We found the soft corals and macroalgae to be more similar in a degraded habitat (Fig. 3C; Suppl. Tables 2 and 3), which may result from their increased acquisition in a degraded habitat (Fig. 2E). As the soft corals acquired more than the macroalgae (and our sponge) (Fig. 2C), we hypothesise that this increased similarity is predominantly due to the exchange of microorganisms from the macroalgae (and the sponge) to the soft corals, which aligns with the observed increase in microbiome richness of *Sinularia* sp. in a degraded habitat. On the other hand, the stony coral *M. digitata* and the sponge *H. cnidata* became more acquirer-like (Fig. 2E) and thus more similar in a biodiverse habitat (Fig. 3C; Suppl. Tables 2 and 3). Comparing patterns in similarity to the number of shared ASVs, we found the sponge’s connectivity to other hosts to be stronger from a microbial member perspective (Fig. 3D; Suppl. Tables 2 and 3).

Similarly, we compared the exuded microbiomes. Even though reef complexity did not significantly influence the level of exudation (Fig. 2D), we found changes in microbiome similarity and member identity between holobionts due to habitat complexity (Fig. 3E and F; Suppl. Tables 2 and 3). With the exception of the stony corals, the exuded microbiomes (and likely the transient interactions between holobionts and nonhost-associated microorganisms) were more similar in a degraded habitat (Fig. 3E; Suppl. Tables 2 and 3), which may be due to a less diverse pool of overall resources (and microorganisms) being available within a less biodiverse coral reef metacommunity [27]. A higher dissimilarity between exuded microbiomes in biodiverse habitats, on the other hand, could be caused by the holobionts exuding metabolites in response to the presence of other host species. Indeed, the soft coral *Xenia* sp. and the macroalgae *Caulerpa* sp. have been reported to produce allelopathic metabolites [34], thereby altering the local resource pool and potentially supporting a different, more diverse microbial community. Interestingly, as fast growers capable of regeneration and propagation, *Xenia* sp. and *Caulerpa* sp. are potentially invasive species [64, 65]. The observed differences in metabolite [34] and microbiome exudation in response to a stony coral–dominated reef metacommunity could be part of their strategy to invade the coral reef community by altering the local environment [27, 34, 66, 67].

## Discussion

**Figure 4:**
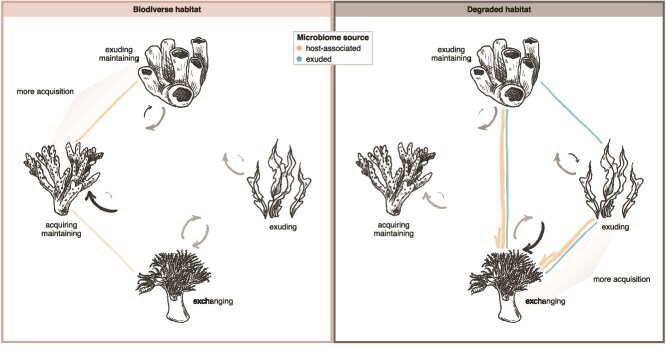
Influence of habitat complexity on the coral reef microbiome. To formulate general hypotheses on the impacts of biodiversity loss on the coral reef microbiome, we summarized and generalized our findings by functional group, contrasting (left) biodiverse and (right) degraded habitats. Bent arrows indicate exudation (arrows pointing away from holobionts) and acquisition (arrows pointing towards holobionts), with size representing the relative magnitude and the colour black highlighting processes that increased in magnitude in the respective habitat complexity. Overall, our results suggest that stony corals (left) are maintaining or acquiring, soft corals (bottom) exchanging, macroalgae (right) exuding, and sponges (top) exuding or maintaining microbiome members. In biodiverse habitats, stony corals and sponges seem to acquire more (or equal amounts of) microorganisms from the environment compared to their counterparts in degraded habitats, while soft corals and macroalgae acquire less. Coloured lines linking the holobionts represent the strongest microbial associations in the respective habitat complexity, with width representing the relative magnitude. Arrowheads indicate the hypothesized directionality of the association where possible. Based on our results, we hypothesize that in biodiverse habitats, stony corals interact more with soft corals and sponges than in degraded habitats. On the other hand, soft corals, macroalgae, and sponges microbially interact more in habitats without stony coral dominance.

To integrate microorganisms into our ecosystem-level understanding of coral reefs, we explored the microbial connections among key benthic reef organisms—stony corals, soft corals, macroalgae, and sponges—and how these links influence and are influenced by the coral reef metacommunity. In our experimental coral reef system, we revealed cascading effects of reducing habitat complexity as a proxy for biodiversity loss from the macro- to the micro-level. We demonstrated that the hosts exhibit species- and functional-group-specific microbial dynamics with distinct patterns of how they interact with each other and the environment, specifically by exuding, maintaining, acquiring, or exchanging microbiome members (Fig. 4). Of these processes, which we characterised descriptively rather than quantitatively, habitat complexity primarily influenced microbial acquisition. Overall, our results indicate that a biodiverse compared to a degraded habitat promotes more microbial acquisition in stony corals and sponges and less in soft corals and macroalgae (Fig. 4). This suggests that the coral reef metacommunity influences the holobionts’ microbiome flexibility—a means to rapidly respond to environmental changes [18, 60], critical in the face of the climate crisis—which, in turn, could determine the long-term success of microbially assisted strategies to tolerate stress.

Additionally, we found the metacommunity to shape the networks within the coral reef microbiome. From a biodiverse to a degraded habitat, the microbial associations linking stony corals to soft corals and sponges became less influential, while tripartite connectivity emerged between soft corals, macroalgae, and sponges (Fig. 4). This connectivity aligns with known positive feedback loops centering around dissolved organic matter that foster the growth of macroalgae and sponges, as well as free-living heterotrophic and pathogenic microbial communities at the expense of stony corals [33, 68], suggesting that microbial links between the holobionts contribute to these feedback loops and host-level community shifts.

Our controlled experimental system, while abstracted from natural coral reef ecosystems [69], served as an effective proxy for hypothesis testing, enabled precise manipulation of host biodiversity, and isolated exudation processes, laying the groundwork for advancing our understanding of natural systems. While we recognize that increased sequencing depth could further enhance the resolution of microbial processes, by studying exudation in isolation, we ensured precise tracking of the exuded microbiome back to its holobiont; however, future studies should explore how interactions with other holobionts during incubation modulate exudation. Our framework assumes that during the two-hour incubation (Materials and Methods), exudation did not reduce microbial richness or diversity within the holobiont and that microbial turnover was negligible. Future studies with longitudinal sampling could explore temporal stability, succession, or turnover in microbial communities, providing deeper insights into their dynamics under varying habitat conditions. In parallel, complementary approaches such as iCAMP [70] could be employed to more explicitly quantify the relative contributions of deterministic and stochastic processes shaping community assembly, offering a phylogenetically informed lens into microbiome dynamics. While our species selection was constrained by experimental feasibility, it allowed targeted insights into microbial dynamics driven by representative species of key coral reef functional groups.

Despite this limited scope, by merging the effects of the coral reef metacommunity at the local (within a single holobiont) and community scale (within multiple holobionts), our results demonstrate how biodiversity loss impacts the coral reef microbiome beyond the boundary of individual hosts. As this could disrupt essential microbial functions that support holobiont and ecosystem health [2, 16], which future studies integrating metagenomic or metatranscriptomic approaches could elucidate, our findings highlight the need to move beyond individual holobionts and consider a metacommunity perspective [71] in conserving and restoring coral reefs to foster a resilient, interconnected microbiome network.

As scientists warn that the loss of microorganism diversity parallels the loss of host biodiversity and that by overlooking microbial life, we risk host and ecosystem collapse [72], we provide empirical evidence for such intricacies within the coral reef microbiome and their links to host–biodiversity loss. However, instead of contemplating what we stand to lose, we encourage a focus on harnessing the enormous potential of host-associated microbiomes [73, 74] to mitigate climate change [75], achieve the Sustainable Development Goals [76], and safeguard the ecosystem functions and services coral reefs provide [77, 78].

## Supplementary Material

Supplementary_Information_ycaf097

Supplementary_Table_1_ycaf097

Supplementary_Table_2_ycaf097

Supplementary_Table_3_ycaf097

Supplementary_Table_4_ycaf097

Supplementary_Table_5_ycaf097

## Data Availability

Raw data from this study will be available from the European Nucleotide Archive as BioProject PRJEB82492. Analysis files and metadata are available on Zenodo (https://zenodo.org/records/14131612).

## Code availability

All code for analyses and figures presented here is accessible through GitHub (https://github.com/SushiLab/coralmicrobiome-hostbiodiversity) and archived on Zenodo (https://zenodo.org/records/14146096).
