# Supplementary material for: Host-level biodiversity shapes the dynamics and networks within the coral reef microbiome
Source: ISME Commun. 2025 Jun 5;5(1):ycaf097. doi: 10.1093/ismeco/ycaf097 (PMC12192423; doi:10.1093/ismeco/ycaf097)
Supplement: Supplementary_Information_ycaf097 [file supplementary_information_ycaf097.pdf]

Supplementary Information

for

Host-level biodiversity shapes the dynamics and networks within the coral reef microbiome

Fabienne Wiederkehr[1], Kara Elena Engelhardt[2], Jana Vetter[2], Hans-Joachim Ruscheweyh[1], Guillem Salazar[1], James O'Brien[1], Taylor Priest[1], Maren Ziegler[2,*], Shinichi Sunagawa[1,*]

[1]Department of Biology, Institute of Microbiology and Swiss Institute of Bioinformatics, ETH Zürich, 8093 Zürich, Switzerland

[2]Department of Animal Ecology & Systematics, Justus Liebig University Giessen, 35392 Giessen, Germany

Supplementary Information

Members of the microbiome that drive the observed community changes might induce stress [1] or promote physiological acclimatisation [2] to the habitat. To elucidate such microbial indicators of change due to habitat complexity (Suppl. Table 5), we employed a differential abundance analysis using ANCOM-BC2 (downloaded from https://github.com/FrederickHuangLin/ANCOMBC/tree/bugfix/R on 17/02/2024) [3]. We automated the removal of taxa with zero variance errors and chose the following parameters for ancombc2: prv_cut=0.25 and struc_zero=TRUE. We determined relatedness between differentially abundant ASVs by aligning sequences using MUSCLE and building a maximum-likelihood tree with the best performing model (general time-reversible discrete gamma model and invariant sites) using R packages msa (v1.36.1) [4] and phangorn (v2.12.1) [5]. Interestingly, only three ASVs were differentially abundant in the host-associated microbiomes (*Winogradskyella* sp. (Bacteroidia) in *Sinularia* sp., Microcystaceae sp. (Cyanobacteria) in *Xenia* sp., and *Anderseniella* sp. (Proteobacteria) in *Caulerpa* sp.) (Suppl. Fig. 6a; Suppl. Fig. 7a). On the other hand, we found 93 differentially abundant ASVs in the exuded microbiomes (amongst which were Subgroup 10 sp. (Acidobacteriota) in *M. digitata* and *Aquibacter* sp. (Bacteroidia) in *Sinularia* sp.) (Suppl. Fig. 6b), suggesting that in terms of abundance, exuded microorganisms respond more to changes in habitat complexity than host-associated ones. The exuded microbiome of *Caulerpa* sp. was particularly rich in differentially abundant ASVs (mostly Planctomycetota spp., Bacteroidota spp., and Proteobacteria spp. and almost exclusively more abundant in a biodiverse habitat), which aligns with the observed overall higher dissimilarity to all other holobionts (Fig. 4e), indicating that the association between *Caulerpa* sp. and these ASVs is only favourable for or supported (through allelopathic metabolites [6]) by the holobiont in a biodiverse environment.

To elucidate how compositional shifts in microbiomes due to habitat complexity may translate to functional changes, we selected two closely related and differentially abundant ASVs of *Sinularia* sp. and compared their predicted functional profiles using PICRUSt2 (v2.5.2) [7],

iterated 50 times, calculated the mean of the predicted KO abundance and unstratified metagenome files, and reclassified the pathways with the KEGG database mapping (2022). We extracted pathways associated with metabolism and removed global and overview maps. *Winogradskyella* sp. (more abundant in the host-associated microbiome in a degraded habitat) and *Aquibacter* sp. (more abundant in the exuded microbiome in a biodiverse habitat) differed in seven pathways related to lipid metabolism, xenobiotics biodegradation and metabolism, and biosynthesis of other secondary metabolites (Suppl. Fig. 7b). The production of secondary metabolites, which can include bioactive compounds, may help maintain holobiont homeostasis [8] and fend off pathogens, predators, and competitors [9, 10]. Indeed, we recently uncovered that coral reef microbiomes exhibit a rich biosynthetic potential [11]. Specifically, we found Subgroup 10 sp. [11] (Acidobacteriota, prevalent in three reef-building corals across the Pacific Ocean basin), which we here found significantly more abundant in the stony coral *M. digitata*'s exuded microbiome in a degraded habitat, to encode a large repertoire of bioactive compound synthesis pathways.

Our analyses suggest that abundance shifts in the microbiome of coral reef hosts, driven by changes in habitat complexity, are species-specific and strongly influence the exuded microbiome. By determining the available resources, the habitat might favour microorganisms capable of assimilating these resources and thus support them in higher numbers [12]. Therefore, leveraging holobiont–habitat interactions may enhance coral reef health and advance bioprospecting by stimulating the production of specific metabolites [6] and fostering microorganisms of interest.

Supplementary Tables

Supplementary Table 1 - Statistical testing of pairwise differences in community composition

Supplementary Table 2 - Statistical testing of alpha diversity differences

Supplementary Table 3 - Microbial community- and member-based networks

Supplementary Table 4 - Statistical testing of changes within networks

Supplementary Table 5 - Differentially abundant ASVs

Supplementary Figures

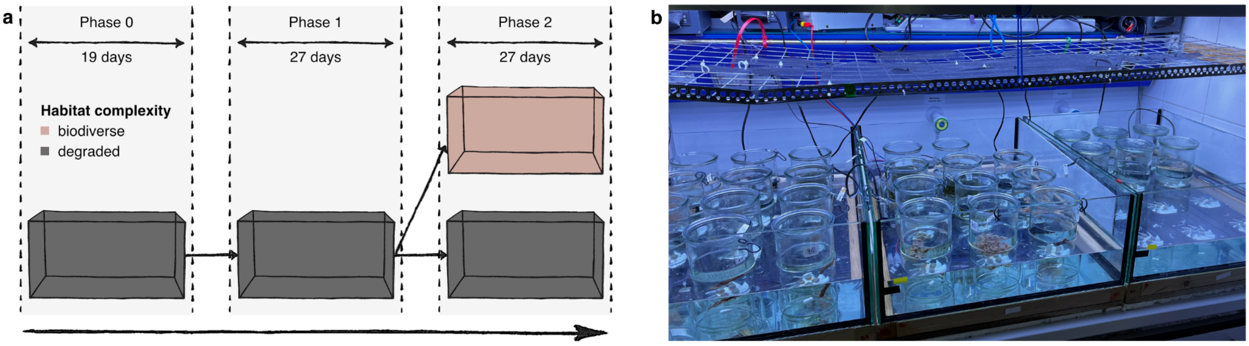

Supplementary Figure 1. Experimental design, sampling regime, and incubation set-up

(a) We conducted a three-month experiment and collected samples after 10 to 13 days (phase 0), after 37 to 40 days (phase 1), and after 64 to 67 days (phase 2, 18 to 21 days after transferring to the respective habitat). After a 46-day acclimatisation period, during which all fragments (n=105) were kept in tanks with degraded complexity containing either only one or all seven of the studied host species, we maintained one part of the fragments (n=63) in these tanks. The remaining fragments (n=42) were transferred to a biodiverse tank, which, in addition to the seven studied host species, included sediment, seagrass, fish, and a diverse benthic community dominated by stony corals. (b) To collect the exuded microbiome, we placed the fragments in incubation jars for 2 h on a multi-point stirring plate [13].

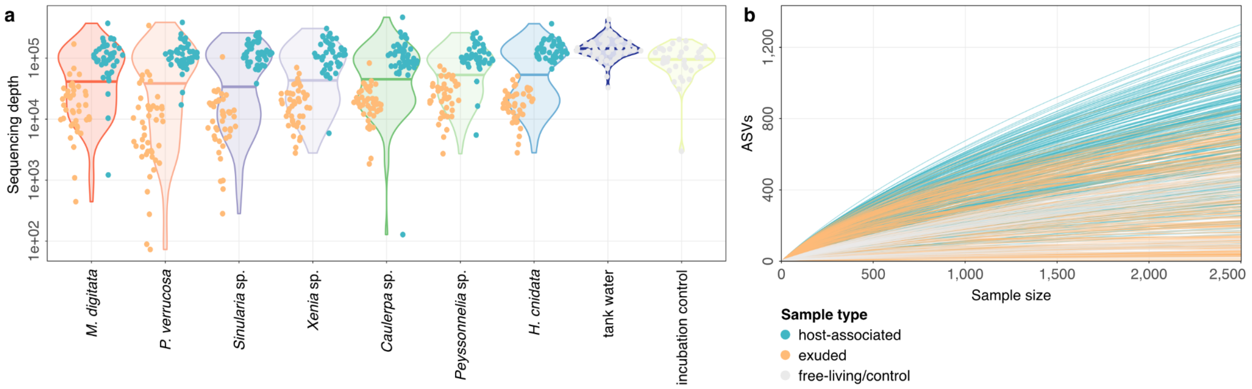

Supplementary Figure 2. Sequencing depth

(a) The sequencing depth was 20,846 ± 24,822 for host-associated samples (sequenced on a MiSeq) and 126,633 ± 62,299 for Sterivex samples, including exuded, control, and tank microbiomes (sequenced on a NextSeq). (b) Rarefaction curves from one of 50 subsampling files show robust stabilisation of the number of ASVs captured for host-associated and free-living microbiomes. All 50 subsampled files were analysed, with mean metric values confidently applied in subsequent analyses (following [14]).

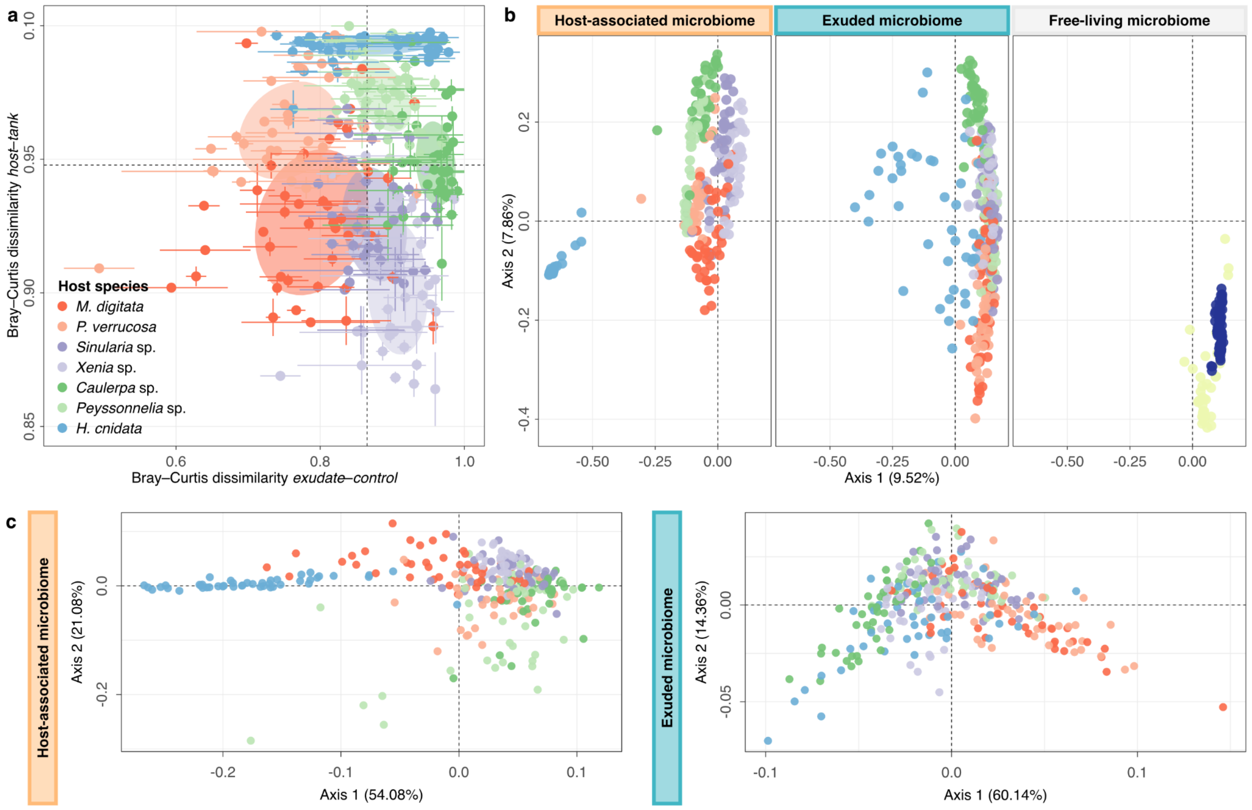

Supplementary Figure 3. Species-specificity in dynamics, composition, and predicted functional potential

(a) To show the variation in dynamics within the coral reef microbiome, we added the standard deviation to each data point, where each point represents the mean distance from a fragment sample to a replicate tank or incubation water (control) sample. (b) A faceted and Bray–Curtis dissimilarity-based Principal Coordinate Analysis (PCoA) on the microbiomes showed species-specificity (host-associated: PERMANOVA, p-value≤0.001, R2=0.31; exuded: PERMANOVA, p-value≤0.001, R2=0.16; all pairwise comparisons significant; Suppl. Table 1) and functional group-specificity (host-associated: PERMANOVA, p-value≤0.001, R2=0.25; exuded: PERMANOVA, p-value≤0.001, R2=0.10; all pairwise comparisons significant; Suppl. Table 1). (c) Using PICRUSt2, a tool for predicting microbiome functional profiles from 16S rRNA data, which relies on reference databases to infer potential functions, we predicted the microbiomes' functional profiles. A Bray–Curtis dissimilarity-based PCoA on the predicted pathways linked to metabolism (KEGG annotations) showed the functional profile to be species-specific (host-associated: PERMANOVA, p-value≤0.001, R2=0.56; exuded: PERMANOVA, p-value≤0.001, R2=0.37; all pairwise comparisons significant, except for exuded M. digitata vs P. verrucosa and exuded Sinularia sp. vs Peyssonnelia sp.) and to cluster the hosts by functional group (host-associated: PERMANOVA, p-value≤0.001, R2=0.49; exuded: PERMANOVA, p-value≤0.001, R2=0.17; all pairwise comparisons significant).

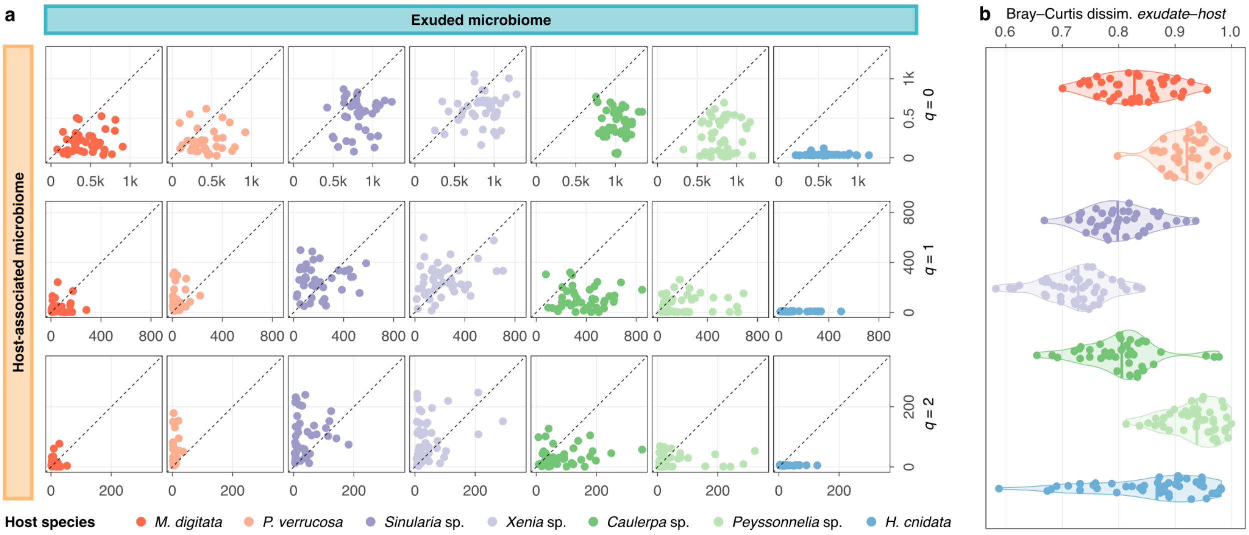

Supplementary Figure 4. Non-random exudation based on microbiome richness, diversity, and dissimilarity

(a) By correlating Hill numbers of orders q=0 (species richness; Fig. 3a), q=1 (exponential of Shannon's entropy index; here), and q=2 (inverse of Simpson's concentration index; Fig. 3b) of the exuded and host-associated microbiomes, we found that alpha diversity diverged most when emphasising abundant microbial community members. Dashed lines are the diagonals, which means equal diversity in host-associated and exuded microbiomes. (b) Comparisons of exuded and corresponding host-associated microbiomes using Bray–Curtis dissimilarities may not solely indicate non-random exudation but could also reflect the presence of microorganisms from sources beyond the studied host-associated microbiomes.

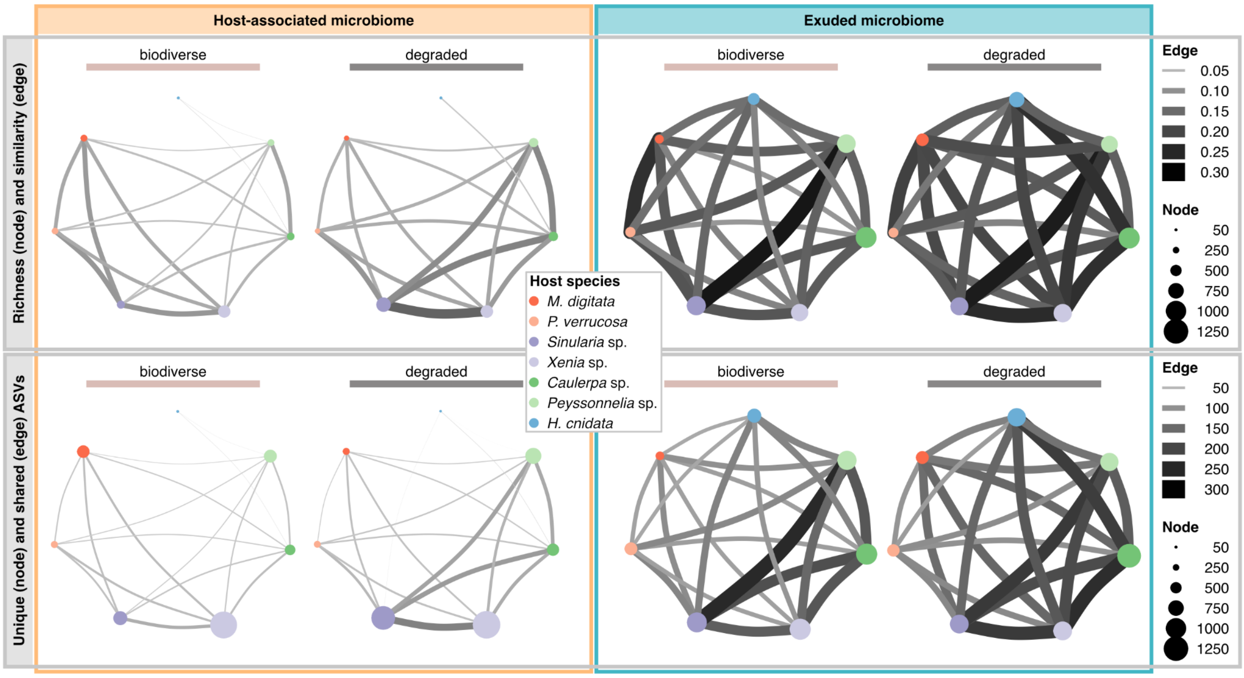

Supplementary Figure 5. Microbial links between coral reef holobionts

We examined (top) microbiome similarity between (edges; Bray–Curtis dissimilarity) and richness of (nodes; Hill q=0) and (bottom) numbers of shared ASVs between (edges) and unique ASVs of (nodes) coral reef holobionts in (left) host-associated and (right) exuded microbiomes. Node size and edge width indicate the magnitude of the interaction. The exuded microbiomes exhibited greater similarities and number of shared ASVs compared to the host-associated microbiomes. Among host-associated microbiomes, stony and soft corals showed the highest similarity in a biodiverse habitat. However, in a degraded habitat, soft corals and macroalgae were more similar, suggesting that changes in the coral reef metacommunity lead to corresponding shifts at the microbial level.

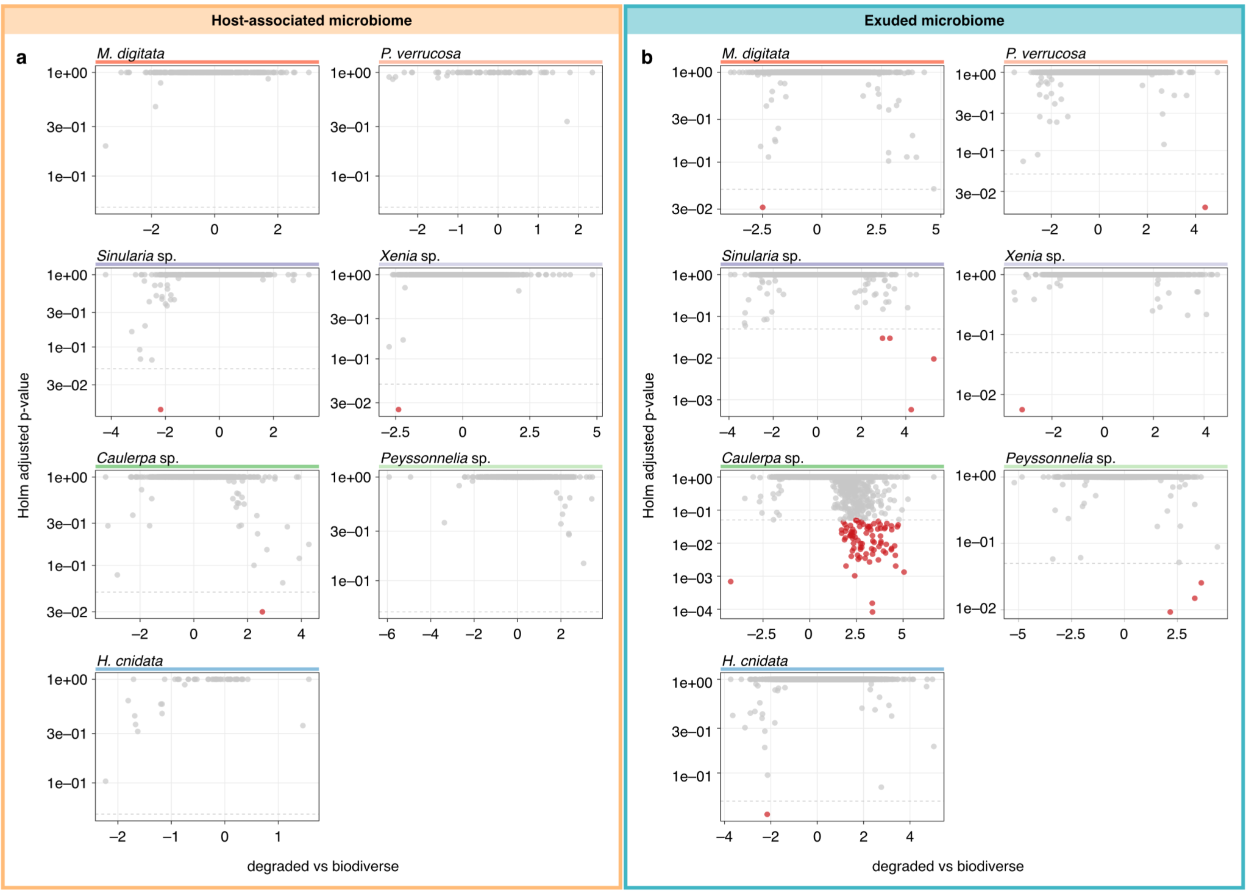

Supplementary Figure 6. Volcano plot displaying differentially abundant ASVs between degraded and biodiverse habitats

We identified the microbiome members that changed significantly in their abundance due to habitat complexity in the (a) host-associated and (b) exuded microbiomes by host species using ANCOM-BC2 [3]. Each point represents an individual ASV, with the x-axis indicating $\log_e$-fold change and the y-axis representing the $\log_{10}$-adjusted p-value. ASVs with a significant adjusted p-value (<0.05) are highlighted in red, indicating differential abundance. ASVs with positive $\log_e$-fold changes are enriched in a biodiverse habitat, while those with negative values are more abundant in a degraded habitat.

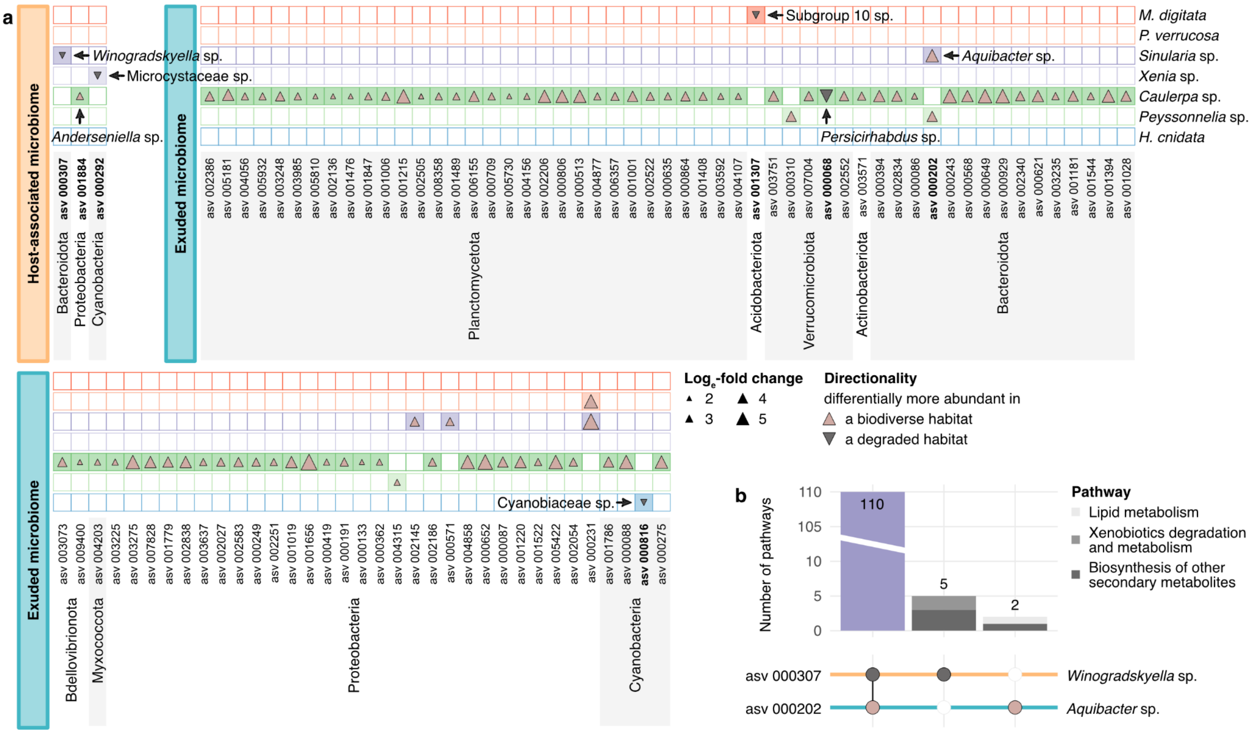

Supplementary Figure 7. Influence of habitat complexity on the abundance of microbial community members

(a) We identified the microbiome members that changed significantly in their abundance due to habitat complexity using ANCOM-BC2 [3] (here, we only show ASVs with taxonomic annotation at the phylum level; Suppl. Fig. 8; Suppl. Table 5). In the host-associated microbiomes, only three ASVs were differentially abundant (in a biodiverse habitat, *Winogradskyella* sp. (Bacteroidia) and a member of the family Microcystaceae (Cyanobacteria) were less abundant in the soft corals *Sinularia* sp. and *Xenia* sp., respectively, and *Anderseniella* sp. (Proteobacteria) was more abundant in the macroalgae *Caulerpa* sp.), while many more ASVs were differentially abundant in the exuded microbiomes (most of which associated with *Caulerpa* sp.). Only three of those ASVs were less abundant in a biodiverse habitat (Subgroup 10 sp. in *M. digitata*, Cyanobiaceae sp. in *H. cnidata*, and *Persicirhabdus* sp. in *Caulerpa* sp.). (b) Using PICRUSt2 [7], we predicted functional profiles. By comparing the closely related *Winogradskyella* sp. (less abundant in a biodiverse habitat and linked to the host-associated microbiome) and *Aquibacter* sp. (more abundant and linked to the exuded microbiome), both associated with *Sinularia* sp., we identified seven pathways related to lipid metabolism, xenobiotics biodegradation and metabolism, and biosynthesis of other secondary metabolites in which the ASVs differed.

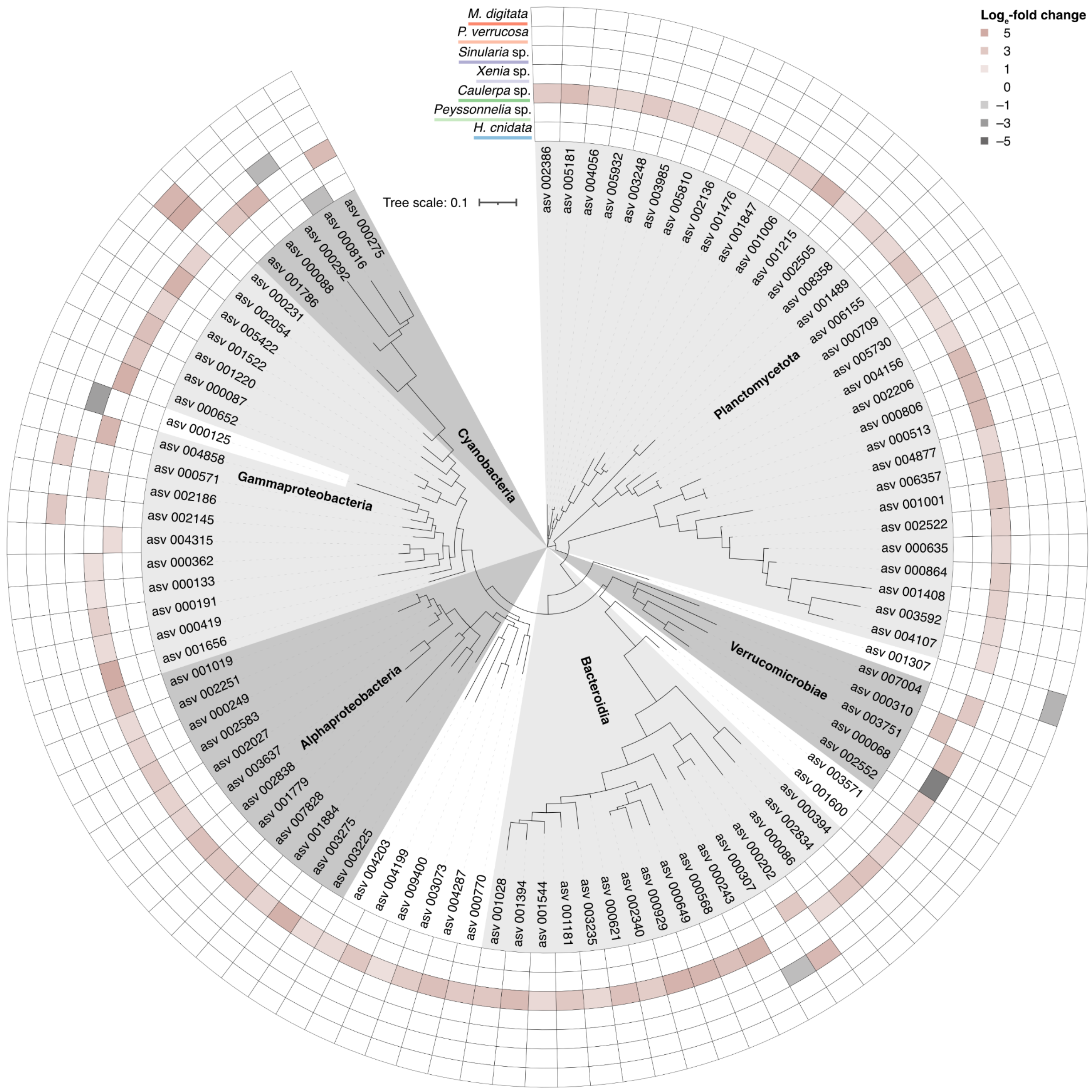

Supplementary Figure 8. Phylogenetic tree of differentially abundant ASVs based on maximum-likelihood analysis

We constructed a phylogenetic tree using MUSCLE for sequence alignment and the best-fit general time-reversible model with discrete gamma distribution and invariant sites (Suppl. Table 5). The relatedness between ASVs is represented by branch lengths, with the tree scale indicating evolutionary distance. The rings surrounding the tree indicate the log$_e$-fold change for each ASV by host species, with positive log$_e$-fold changes indicating higher abundance in a biodiverse habitat, while negative values represent enrichment in a degraded habitat.

References

1. Pogoreutz C, Rädecker N, Cárdenas A, Gärdes A, Wild C, Voolstra CR. Dominance of *Endozoicomonas* bacteria throughout coral bleaching and mortality suggests structural inflexibility of the *Pocillopora verrucosa* microbiome. *Ecol Evol* 2018; **8**: 2240–2252.
2. Voolstra CR, Ziegler M. Adapting with microbial help: Microbiome flexibility facilitates rapid responses to environmental change. *BioEssays* 2020; **42**: e2000004.
3. Lin H, Peddada SD. Multigroup analysis of compositions of microbiomes with covariate adjustments and repeated measures. *Nat Methods* 2024; **21**: 83–91.
4. Bodenhofer U, Bonatesta E, Horejš-Kainrath C, Hochreiter S. msa: An R package for multiple sequence alignment. *Bioinformatics* 2015; **31**: 3997–3999.
5. Schliep KP. phangorn: Phylogenetic analysis in R. *Bioinformatics* 2011; **27**: 592–593.
6. Engelhardt KE, Vetter J, Wiederkehr F, Hartwig C, Klinkenbuß D, Sunagawa S, et al. Biodiversity affects the exometabolomes of four benthic functional groups in coral reefs. Doi:10.21203/rs.3.rs-5083584/v1
7. Douglas GM, Maffei VJ, Zaneveld JR, Yurgel SN, Brown JR, Taylor CM, et al. PICRUSt2 for prediction of metagenome functions. *Nat Biotechnol* 2020; **38**: 685–688.
8. Zhang Y, Ling J, Yang Q, Wen C, Yan Q, Sun H, et al. The functional gene composition and metabolic potential of coral-associated microbial communities. *Sci Rep* 2015; **5**: 16191.
9. Ritchie KB. Regulation of microbial populations by coral surface mucus and mucus-associated bacteria. *Mar Ecol Prog Ser* 2006; **322**: 1–14.
10. Modolon F, Barno AR, Villela HDM, Peixoto RS. Ecological and biotechnological importance of secondary metabolites produced by coral-associated bacteria. *J Appl Microbiol* 2020; **129**: 1441–1457.
11. Paoli L, Wiederkehr F, Ruscheweyh H-J, Miravet-Verde S, Bistolas KSI, Sawyer T, et al. Genome-resolved diversity and biosynthetic potential of the coral reef microbiome. *bioRxiv*: 2024.08.18.608444.

12. Haas AF, Fairoz MFM, Kelly LW, Nelson CE, Dinsdale EA, Edwards RA, et al. Global microbialization of coral reefs. *Nat Microbiol* 2016; **1**: 16042.

13. Rades M, Schubert P, Ziegler M, Kröckel M, Reichert J. Building plan for a temperature controlled multi-point stirring incubator. 2022. Justus Liebig Universität Gießen.

14. Schloss PD. Rarefaction is currently the best approach to control for uneven sequencing effort in amplicon sequence analyses. *mSphere* 2024; 9: e0035423.
